# The Microstructure, Mechanical, and Friction-Wear Properties of Boron Carbide-Based Composites with TiB_2_ and SiC Formed In Situ by Reactive Spark Plasma Sintering

**DOI:** 10.3390/ma17102379

**Published:** 2024-05-16

**Authors:** Agnieszka Twardowska, Marcin Kowalski

**Affiliations:** Institute of Technology, University of the National Education Commission, 2 Podchorazych, 30-084 Krakow, Poland; marcin.kowalski@uken.krakow.pl

**Keywords:** boron carbide B_4_C, TiB_2_, SPS, microstructure, properties, wear

## Abstract

The paper presents the influence of the temperature of the sintering process on the microstructure and selected properties of boron carbide/TiB_2_/SiC composites obtained in situ by spark plasma sintering (SPS). The homogeneous mixture of boron carbide and 5% vol. Ti_5_Si_3_ micropowders were used as the initial material. Spark plasma sintering was conducted at 1700 °C, 1800 °C, and 1900 °C for 10 min after the initial pressing at 35 MPa. The heating and cooling rate was 200 °C/min. The obtained boron carbide composites were subjected to density measurement, an analysis of the chemical and phase composition, microstructure examination, and dry friction-wear tests in ball-on-disc geometry using WC as a counterpart material. The phase compositions of the produced composites differed from the composition of the initial powder mixture. Instead of titanium silicide, two new phases appeared: TiB_2_ and SiC. The complete disappearance of Ti_5_Si_3_ was accompanied by a decrease in the boron carbide content of the stoichiometry formula B_13_C_2_ and an increase in the content of TiB_2_, while the SiC content was almost constant. The relative density of the obtained boron carbide composites, as well as their hardness and resistance to wear, increased with the sintering temperature and TiB_2_ content. Unfortunately, the reactions occurring during sintering did not allow us to obtain composites with high density and hardness. The relative density was 76–85.2% of the theoretical one, while the Vickers hardness was in the range of 4–12 GPa. The mechanism wear of boron carbide composites tested in friction contact with WC was abrasive. The volumetric wear rate (Wv) of composites decreased with increasing sintering temperature and TiB_2_ content. The average value of coefficient of friction (CoF) was in the range of 0.54–0.61, i.e., it did not differ significantly from the value for B_4_C sinters.

## 1. Introduction

Boron carbide with the nominal stoichiometric formula B_4_C is the third hardest single-phase materials known, after diamond and cubic boron nitride. In addition to its high hardness (29 ÷ 35 HV), its attractiveness is provided by its low density (2.52 g/cm^3^), high melting point (2450 °C), high modulus of elasticity E (achieving value of 470 GPa), high resistance to wear, thermal stability, and high corrosion resistance [1,2,3,4,5,6,7,8]. Such a unique set of properties makes this material widely used in the machinery and cutting tool industry (abrasive materials, wear-resistant components), armaments (lightweight armors), and aerospace (temperature-resistant components of jet engines). Boron carbide is also characterized by having one of the highest neutron absorptions. For this reason, sinters made of this carbide are used in the nuclear industry (controlling rods [1,2]. It is also tested as a potential candidate for use in boron neutron therapy [9]. However, there are some limitations that prevent the wider use of this carbide, the most important of which are its low fracture toughness and poor sinterability. Both are related to the covalent bonds prevailing in boron carbide [10,11]. Obtaining dense boron carbide sinters by hot-pressing (HP) or pressureless sintering (PS) is difficult and usually requires a temperature of sintering above 2200 °C and a relatively long time of processing, which leads to unwanted grain growth and quality deterioration of the resulting products. Additionally, high temperature and long times of sintering lead to low mechanical properties and a high cost of production. To solve these problems, various types of second-phase additions have been introduced to boron carbide-based composites as strengthening phases, among them TiB_2_ [11,12,13], MoSi_2_ [14], Si [10], ZrO_2_ [13,14], SiC [11,14], Al_2_O_3_ [15] and Ti-Al intermetallic [16]. In particular, TiB_2_ and SiC are desired second-phase additions for boron carbide ceramics, because they exhibit high hardness and low density, and simultaneously improve the sinterability and toughness [15]. TiB_2_ addition is also helpful in reducing grain growth during sintering [17,18]. Due to the beneficial effects of TiB_2_ and SiC as second-phase additives in boron carbide-based composites, attempts have been made to produce material with improved properties, using different methods. So far, the most frequently used have been based on the direct mixing of powders in the above-mentioned phases with different proportions. Recently, much attention has been paid to obtaining the desired phase composition of boron carbide-based composites by reactive sintering of boron carbide with the use of different secondary precursors. For example, dense B_4_C-SiC-TiB_2_ composites were obtained through reactive hot-pressing sintering using the additive titanium silicon carbide Ti_3_SiC_2_ [10]. In the other cases, B_4_C-SiC-TiB_2_, with its superior combined mechanical properties, was obtained by adding 5% of (Ti_3_SiC_2_ + Si) sintering aid [18] or by reactive hot-pressing of B_4_C, with ZrO_2_ addition [19], TiC and Si [20,21]. Wang et al. [22] successfully fabricated B_4_C-SiC-TiB_2_ composites in situ by an SPS method using B_4_C and TiSi_2_ as starting materials. They found that TiSi_2_ serves as a transient liquid phase, because of this, the composite is highly densified [22]. Meanwhile, Kozien et al. [23] analyzed pressureless sintering processes of B_4_C powders with the addition of Ti_5_Si_3_. They showed that reactive sintering of B_4_C and Ti_5_Si_3_ powders leads to the complete disappearance of this silicide and the formation of TiB_2_ and SiC phases. The resulting B_4_C-SiC-TiB_2_ composites had higher mechanical properties than those obtained with a similar addition of TiSi_2_. Based on the results of the work of these authors, this work attempts to obtain dense composites based on boron carbide by reactive sintering of boron carbide with the addition of titanium silicide Ti_5_Si_3_, but using the spark plasma sintering method (SPS), which, to our knowledge, has not been done so far. Spark plasma sintering is more beneficial for the microstructure and properties of the produced materials due to much shorter sintering times, lower temperatures required to densify materials, and faster heating and cooling rates (in comparison to “conventional” methods of sintering as HP), which allows us to avoid or significantly reduce grain growth in the resulting composites. Moreover, this method allows us to obtain a high-purity material, free of oxide contamination by eliminating the oxide layer from the surface of boron carbide powder during sintering. In our case, boron carbide composites were obtained by reactive SPS sintering of boron carbide and Ti_5_Si_3_ as starting materials, at 1700 °C, 1800 °C, and 1900 °C for 10 min. The main aim of this work is to investigate the influence of sintering temperatures on chemical and phase composition, microstructure, hardness, and friction-wear properties of the produced composites tested in technically dry friction contact with tungsten carbide.

## 2. Experimental Procedure

### 2.1. Initial Powders and Powder Homogenization

**Figure 1:**
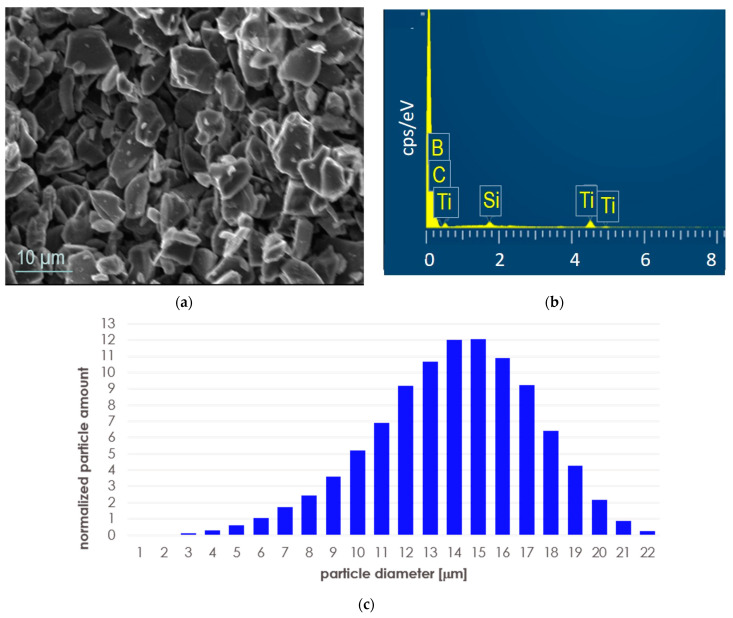
B_4_C/5% vol. Ti_5_Si_3_ powder mixture after homogenization: (**a**) SEM image; (**b**) EDS spectrum taken from the presented area; (**c**) particle size distribution.

As initial powders, we used boron carbide sold by Sigma Aldrich (Darmstadt, Germany) under the catalog designation as B_4_C with a density of 2.52 g/cm^3^ and an average particle size of less than 10 μm. In fact, it contained 99 wt.% boron carbide of stoichiometry B_13_C_2_ and 1 wt.% of graphite C. As a second component, we used titanium silicide Ti_5_Si_3_ (Alfa Aesar, Schiltigheim, France) of a purity of 99.5 wt.% Ti_5_Si_3_ and of 325 mesh. These powders were used to make a mixture containing 95% by volume of boron carbide and 5% by volume of Ti_5_Si_3_. The mixture was homogenized with SpeedMixerTM DAC 400.1 FVZ (Hauschild, Germany) in three stages, using the following parameters: rotational speed 2000 rpm, rotational time 30 s, break time 10 s. An SEM image of the initial powder mixture after homogenization is shown in Figure 1a, with EDS analysis (taken from the whole presented area) in Figure 1b. After homogenization, the powder mixture was analyzed by the DLS technique (dynamic light scattering) using the nanoparticle size analyzer Shimadzu SALD-7500 operating (Kyoto, Japan) at λ = 405 nm (the absorbance was 0.106–0.206, ultrasonic bath, distilled water suspension, 5 min). The grain size distribution in the mixture is shown in Figure 1c and Table 1.

### 2.2. Spark Plasma Sintering

**Figure 2:**
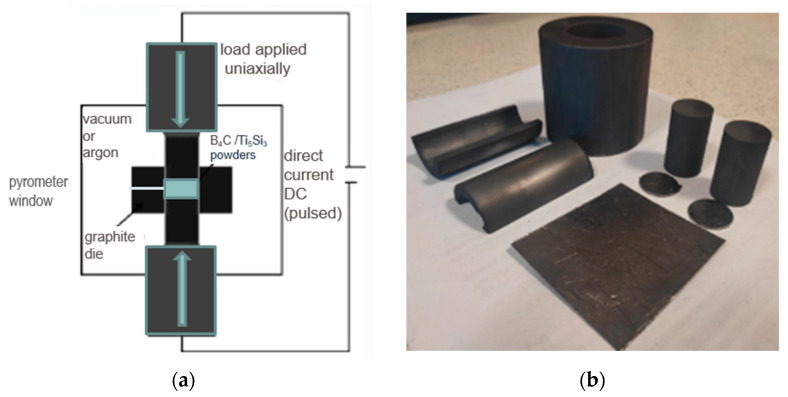
(**a**) Schematic representation of an SPS sintering device. (**b**) Components of the sintering die.

Powder consolidation was realized with SPS Sinter Press Dr. Fritsch LSP-100 (Fellbach, Germany), in a graphite die (Figure 2). The process started with the initial pressing (35 MPa) in a vacuum (5 × 10^−1^ mbar) and heating up to the sintering temperature with a heating rate of 200 °C/min. The temperature was controlled by a pyrometer. After reaching the desired temperature (1700 °C, 1800 °C, and 1900 °C), the sintering process was conducted (in argon) for 10 min, then the furnace system started cooling down at a rate of 200 °C/min. The samples were obtained as discs with a diameter of 20 mm and a height of ~7 mm. Then samples were mechanically ground using Struers (Ballerup, Denmark) MD grinding disc and diamond suspensions (9, 6, and 3 μm) and polished on one side (1 μm). The samples were cleaned in water after each grinding operation, degreased in isopropanol, then ultrasonically cleaned in distilled water for 5 min and dried.

### 2.3. Materials Characterization

The Archimedes immersion method was used for the determination of densities of composites by measuring the difference in the sample’s weight in air and in distilled water (at room temperature conditions). For each composite sample, the average density value was determined (based on three measurements carried out after drying it in a dryer) and related to the theoretical density, calculated according to the rule of mixtures, taking into account the values of the densities of phases identified in the composite by the X-ray diffraction (XRD) method with their content determined by the Rietveld refinement. Phase compositions of composites and initial powders were analyzed by Rigaku MiniFlex X-ray diffractometer (Tokyo, Japan) in Bragg–Brentano geometry using Cu K_α_ radiation (λ = 1.5406 Å, *U* = 30 kV, I = 15 mA). Diffractograms were collected in 2Θ scattering angles ranging from 20° to 90° (step size of 0.02°). Phase identification was carried out according to the ICSD database. The microstructure of composites was observed using scanning confocal microscopy (Olympus 3D LEXT™ OLS5100, Tokyo, Japan) and scanning electron microscopy (SEM, JEOL JSM 6610LV, Tokyo, Japan). During the SEM observations, the chemical composition was analyzed in the selected areas by energy dispersive spectroscopy (EDS). The recorded EDS spectra and element distribution maps were interpreted using Aztec 2.0 software by Oxford Instruments (Abingdon, UK). Vickers hardness (HV1) was measured under a load of 9.81 N, using a NEXUS 4000 hardness tester (INNOVATEST Europe BV, Maastricht, The Netherlands). Ten indentations were made at random locations of the surface of each sample for the calculation of the average value of HV1. Friction-wear tests were performed in ball-on-disc geometry, according to the ISO 20808:2016(E) [24]. Samples were tested in “dry” friction contact with WC, as the counterpart material. Dry tests are intended to create possibly extreme conditions of cooperation for the tested material being in friction contact. Counterpart material (WC) was selected for friction-wear tests due to its high hardness (30 GPa) in comparison to the hardness of the tested composites. Besides, it is the main component of WC-Co cutting tools, in which geometry can be shaped or finished using boron carbide (tools or abrasives). In ball-on-disc tests, the WC balls had a diameter of 3.175 mm and for each try, a new ball was used. Balls were loaded at 5 N, the diameter of the wear track was fixed at 10 mm, and the length of sliding was 1000 m. The linear sliding speed was v = 0.1 m/s. Friction-wear tests were conducted in the air with a humidity of 40–45%, and the average temperature during tests was 21 °C. The extensometer was used for continuous measuring of the friction force during the test. The ratio of the friction force (F_t_) and the applied load (F_n_) was used for friction coefficient calculation. The cross-sectional profile lines of the wear track were measured optically using scanning confocal microscope Olympus 3D LEXT™ OLS5100. The profile lines were collected in four places on its circumference, with distances of 90 degrees. These profiles were used to estimate the cross-sectional area of the track and to calculate the volume of the worn material and the specific wear rate, Wv according to the following equation:

(1) $$W_{v}=\frac{V_{L}}{F_{n}\times L}$$

where:

V_L_—volume of worn material (disc specimen) [m^3^] optically measured;
F_n_—load applied [N];
L—sliding distance [m].

## 3. Results and Discussion

### 3.1. Microstructure

**Figure 3:**
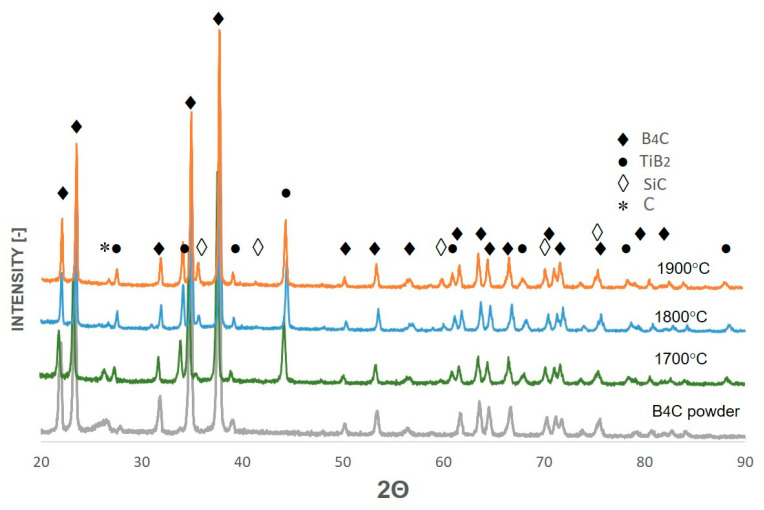
XRD patterns registered in BB geometry for composites obtained from B_4_C/5% vol. Ti_5_Si_3_ powder with indexed peak positions for identified phases.

Figure 3 shows the X-ray diffraction patterns registered for composites sintered from boron carbide powder with the addition of 5% vol. of Ti_5_Si_3_ powder at 1700 °C, 1800 °C, and 1900 °C. The main phase identified in the produced composites was rhombohedral R3m boron carbide B_13_C_2_ (01-071-0108). The B-C equilibrium system is the subject of ongoing research and discussion. According to the widely accepted binary B-C phase diagram [23], boron carbide, which is written under the summary formula B_4_C, exists as the most thermodynamically stable phase in a relatively wide range of carbon contents, i.e., from approximately 8.5 to 20.5 at %C. Boron carbide with a stoichiometry formula B_13_C_2_ falls within this range. B_13_C_2_ was the only boron carbide identified in the initial powder sold by Sigma Aldrich (Saint Louis, USA) as B_4_C (see Figure 2, grey line). The structural atomic formula of stoichiometric B_4_C is (B12) CCC [24]. In the case of boron-rich B_13_C_2_, the atomic structural formula is (B12) CBC, in which additional one boron atom replaces one carbon atom in the C-C-C chain in the hexagonal unit. The existence of an idealized carbon-rich compound B_4_C was questioned; hence, various variants of the B-C phase diagrams were proposed, with numerous phases with variable stoichiometry present in the low and intermediate temperature ranges [15,24]. Theoretical investigations by DFT calculations of various configurations of the chain for boron carbide of different stoichiometry indicated that B_13_C_2_ is a stable form of boron carbide in temperatures above 330 °C [25]. Due to the ongoing discussion about the form of the B-C phase diagram and the phases constituting it, for the interpretation of the composition of our samples, we assumed that the boron carbide with stoichiometry B_13_C_2_ identified by us is the boron-rich B_4_C polytype, and therefore in this work it is written in this way, similarly to other authors [23].

The lattice parameters we calculated for boron carbide B_13_C_2_ on the basis on XRD measurements were a = 5.991–5.602 Å and c = 12.071–12.075 Å, with a c/a axial ratio 2.015–2.154, while for the ICCD standard for B_13_C_2_, a = 5.5985 Å and c = 12.0664 Å, with an axial ratio c/a = 2.155. A slight expansion of the lattice parameters of boron carbide identified in composites is consistent with the results obtained for boron-rich boron carbide [25].

No traces of the titanium silicide Ti_5_Si_3_ phase was identified in any of the composites. Instead, two other phases appeared: titanium diboride TiB_2_ (04-010-8469) and 3C-SiC (73-1665). The same changes in phase composition were found by other authors [22] during the sintering of B_4_C with the addition of Ti_5_Si_3_ (in the range of 5–15 wt.% of this addition). Based on the thermodynamic data and theoretical calculations, these authors concluded that the reaction which takes place in the B_4_C and Ti_5_Si_3_ system is Reaction (2):

2Ti_5_Si_3_ + 5B_4_C + C → 10TiB_2_ + 6SiC, with ΔG = −771380 − 35.16∙T (2)

as, for this reaction, the lowest free enthalpy ∆G value was calculated in the temperature range from 298 K to 2698 K [22].

The lattice parameters calculated for TiB_2_ (hexagonal close-packed; hcp) were a = 3.0305 Å and c = 3.2339 Å, which are close to ICCD data: a = 3.02 Å and c = 3.22 Å for this boride. In the case of silicon carbide 3C-SiC, the lattice parameter was a = 4.3571 Å, while for the ICCD standard, it was a = 4.358 Å. The presence of trace amounts of graphite (04-015-2432) hcp (a = 2.47 Å, c = 6.863 Å) was also identified in each of the resulting composites. The trace presence of graphite may be related to the graphite punches and matrices used in the SPS process.

The titanium diboride content increased with the sintering temperature from 1 to 2.8 wt.%. The increase in the content of this phase was accompanied by a decrease in the content of boride carbide, while the silicon carbide content was almost constant, i.e., approximately 0.6 wt.%.

**Figure 4:**
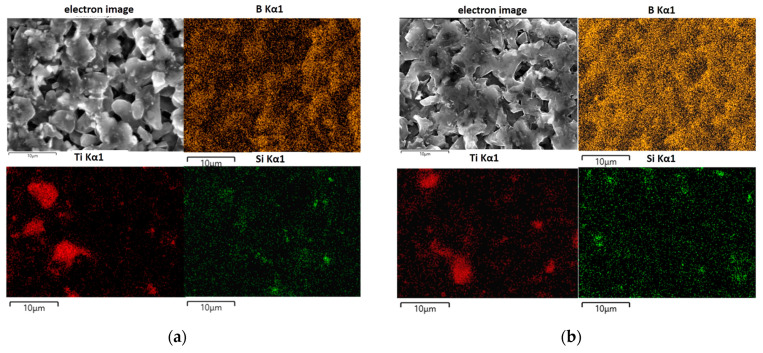
SEM image of the microstructure of B_4_C-TiB_2_-SiC composites with EDS elemental maps of Ti, Si, and B distribution: (**a**) 1800 °C; (**b**) 1900 °C.

Density measurements confirmed the difficulties in obtaining high-density composites (Table 2). Reactive sintering that occurred during sintering did not allow us to obtain the theoretical density even at a relatively high sintering temperature of 1900 °C. The low sinterability of boron carbide comes from a high content of covalent B-C bonds and a low self-diffusivity coefficient. The sinterability of boron carbide is also influenced by the presence of an oxide layer on the surface of the grains, which is difficult to remove and remains in conventionally sintered material as a contaminant. In the case of SPS sintering, oxide layers are not so important for sinterability, because they are quickly destroyed and removed in the initial stages of the process. The maximum value of relative density was ~85% of the theoretical one. The detailed microstructure analysis of B_4_C-TiB_2_-SiC composites we obtained at selected temperatures confirmed incomplete powder consolidation. The composite sintered at 1700 °C was the most porous, and easy to crush. Therefore, this sample was excluded from further research. The grain size did not change significantly after sintering, i.e., the average grain size was ~10 microns. The sintering temperature affected the density of the composites, but also elemental distribution, as shown in Figure 4a,b (EDS maps). The distribution of boron (yellow color) in the composites shows essentially no significant differences in the produced composites. However, there were some differences in the distribution of titanium and silicon. In the composite sintered at 1800 °C, areas enriched in titanium are clearly visible (in red color) with a sharp contrast against the black background, while silicon-rich areas (green color) are of relatively low intensity with blurred contrast at boundaries. In the composite sintered at 1900 °C, the boundaries of areas with increased titanium are blurred, while the boundaries of areas with increased amounts of silicon are more clearly visible and become more intense. Such blurring of the boundaries between areas enriched in the content of individual elements indicates that diffusion processes take place there.

**Figure 5:**
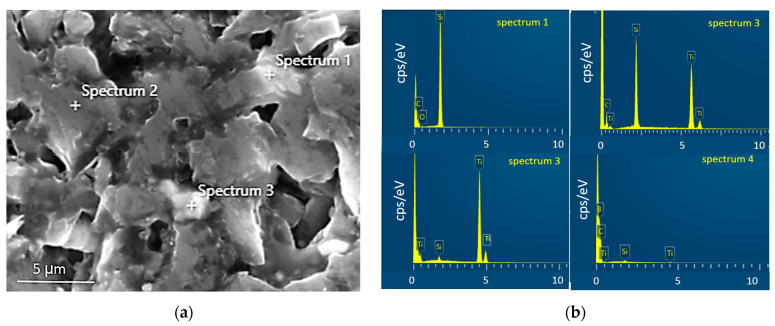
(**a**) The microstructure (SEM image) of the composite sintered at 1900 °C with (**b**) EDS spectra taken from marked area (spectrum 1–3) and whole area (spectrum 4).

Based on these observations and the results of XRD analysis, shown in Figure 4 and Table 2, it can be concluded that a higher sintering temperature (favoring elemental diffusion) resulted in a slightly lower boron carbide content in composites, accompanied by the complete disappearance of the Ti_5_Si_3_ phase to create the TiB_2_ and SiC phases. However, in Figure 5, the presented spectrum EDS (Figure 5b) clearly indicates the presence of titanium silicide in the selected micro-area. The lack of reflections from titanium silicides in registered diffractograms does not mean that they are not present even in trace amounts. It means that their content is beyond the detection capabilities of the XRD method.

### 3.2. Hardness and Resistance to Wear

**Figure 6:**
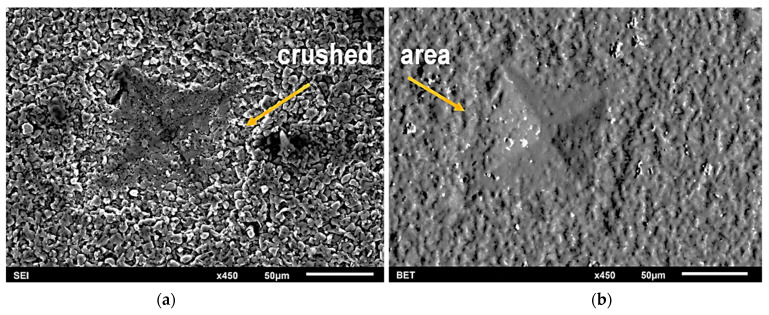
SEM images of the surface of the composite sintered at 1900 °C after hardness measurement in indented area: (**a**) secondary electron image; (**b**) backscattered electron image in topography mode (BET).

The hardness of B_4_C-TiB_2_-SiC composites was in the range of 4–12 GPa, which, compared to the literature values of 34.5-GPa for B_4_C sinters obtained by the SPS method [24], is low. However, similar results for Vickers hardness were reported for B_4_C-TiB_2_-SiC composites obtained by the reactive sintering of B_4_C/Ti_5_Si_3_ powders of the same composition by pressureless sintering [23]. In fact, all composites and starting powders were identified as boron carbide of stoichiometry B_13_C_2_. Boron carbide with stoichiometry B_13_C_2_ has a smaller content of covalent bonds because in the C-C-C chain of the unit cell, one of the carbon atoms is replaced by one boron atom. This may be the reason for the lower hardness of our composites, as we found in our hardness tests. However, such significant discrepancies in hardness are most probably due to the measurement of the diagonals of indentations. It was probably overestimated due to severe chipping of the material. During unloading, the material crumbled strongly at the surface, especially near the external edges of indentations as shown in Figure 6. This behavior of the material was observed during hardness testing regardless of the load. Even when using the nanoindentation method, the results were not shown. For the same sample, results were obtained within a wide range of hardness values with an error exceeding 15% of the average value. The applied load of 9.81 N did not cause the formation of cracking in the corners of the indented area. Internal stresses are considered to be one of the probable causes of such behavior of B_4_C-TiB_2_ composites under the influence of a localized load. During cooling after sintering, internal stresses arise in the phase components (B_4_C and TiB_2_) present in composite materials. The stress levels differ in phase components due to their thermal expansion coefficients. The mismatch of thermal properties causes stresses at the phase interface and in the presence of the load leads to microcrack initiation. Excess strain energy is absorbed into the local disintegration of the material, near the subsurface area of indentation and around it; therefore, cracks do not occur in the corners. Another explanation for microcracking in these composites is related to the partial amorphization of B_4_C, which could be induced by impact pressure. The presence of an amorphous phase was confirmed by HR TEM examinations of boron carbide-sintered samples subjected to ballistic tests [26]. Stress-induced transformation of boron carbide was also reported in static indentation [27], nanoindentation tests [28,29], and scratch tests [30]. This explanation is generally not inconsistent with our observations. The presence of an amorphous phase at the inter-phase boundaries may increase the tendency to brittle cracking, as the amorphous phase is more brittle compared to its crystalline counterpart [4].

**Figure 7:**
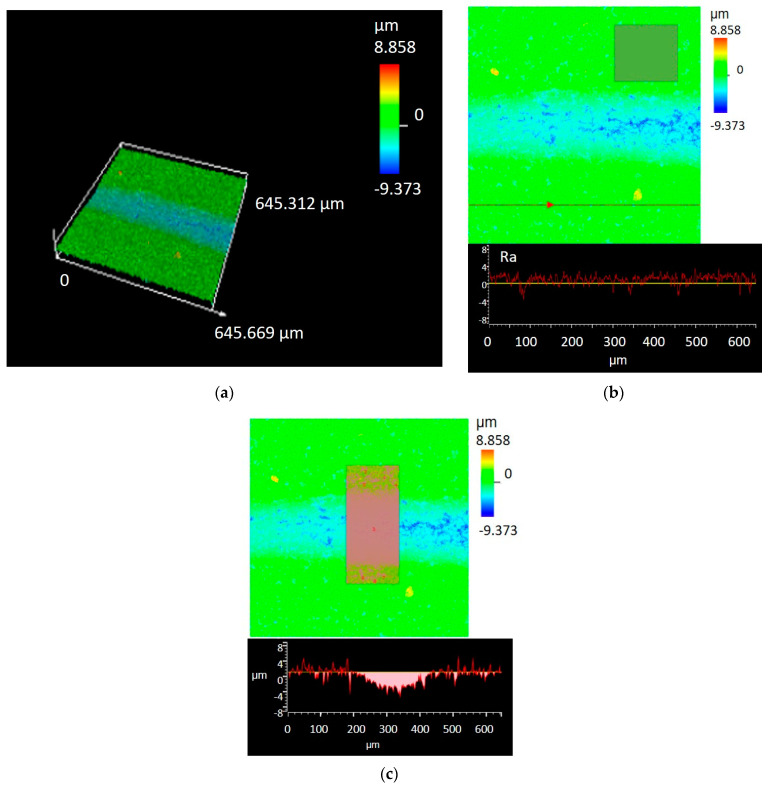
Exemplary results of roughness and volume loss measurement of produced composites by confocal microscopy after ball-on-disc test (in dry sliding with WC ball as a counterpart material): (**a**) surface height 3D map; (**b**) roughness profile; (**c**) surface area of wear track cross-section for volume loss measurement.

**Figure 8:**
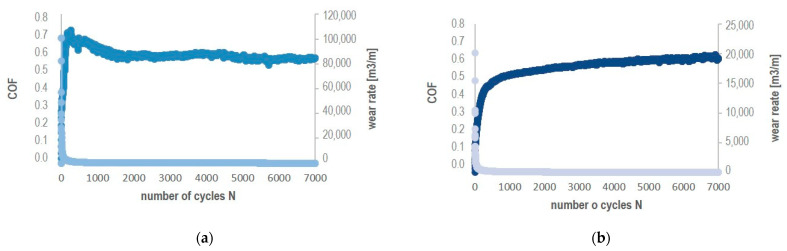
Experimental plots of CoF and wear rate of produced composites, sintered at (**a**) 1800 °C; (**b**) 1900 °C.

The wear rate and friction coefficient CoF values were determined in ball-on-disc test in dry sliding with a WC ball as a counterpart material. The roughness parameters Ra, surface roughness Sa, and volume loss V_L_ were determined by optical measurements using a confocal microscope. The average values of Ra and Sa parameters for samples sintered at 1700 °C, 1800 °C, and 1900 °C were, respectively, 1.6 µm/1.8 µm, 1.5 µm/1.18 µm, and 1.2 µm/1.12 µm. Figure 7a shows a sample image recorded by confocal microscopy from the surface of the sample after the ball-on-disc test, which was used for roughness analysis and volume loss measurement. Figure 7b shows the position of the line used for the Ra profile measurement and the area (beige rectangle) for the surface roughness parameters Sa calculations, while Figure 7c shows the area of the friction path with the marked place (rectangle) from which the volume loss was calculated. The experimental plot for CoF and the wear rate vs. the number of cycles N are given in Figure 8.

**Figure 9:**
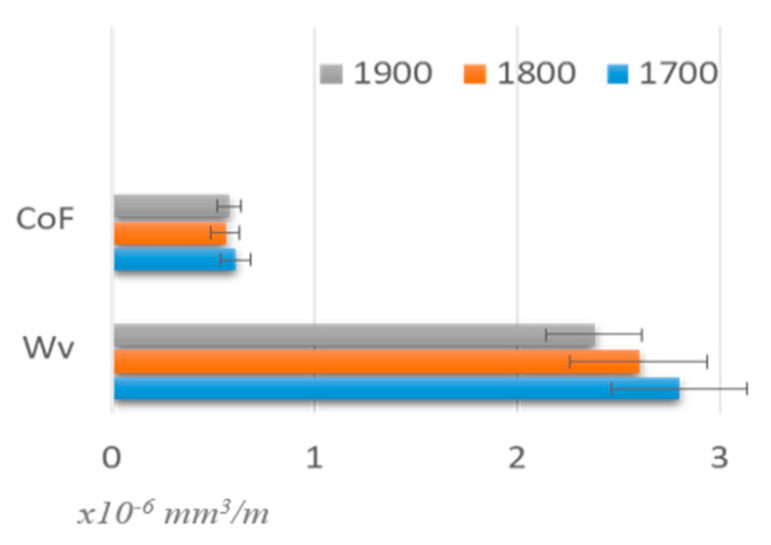
CoF values and volumetric wear indexes (Wv) for boron carbide composites determined in friction-wear test with WC as counterpart material.

According to the CoF plot (Figure 8a, light blue), in the initial stage of the friction-wear test, the value of the friction coefficient increases quickly to 0.71, then slightly decreases to 0.6 (N-1000) and stabilizes at this value to 5000 cycles, then slightly decreases to 0.54. For the composite sintered at 1900 °C (Figure 8b, light green), the course of the CoF/N relationship starts with an initial rapid increase to 0.4, then slightly increases to 0.6, reaching the maximum value of 0.61. Average values for B_4_C-TiB_2_-SiC composites in the whole test were 0.54 and 0.58 for samples sintered at 1800 °C and 1900 °C, respectively. The CoF values generally corresponded to the reference value for the B_4_C sinter (0.6), within the margin of error. Plots for average values for CoF values and volumetric wear indexes (Wv) for B_4_C-TiB_2_-SiC composites tested in friction-wear tests are presented in Figure 9. The B_4_C-TiB_2_-SiC composite sintered at the highest temperature had the lowest value of the friction coefficient CoF and the highest resistance to wear (lowest volumetric wear loss Wv). Both parameters for this sample were slightly better than the reference values for B_4_C sinters. The differences in the values of the CoF and Wv indicators for the tested composites are not large and should be considered in the context of their relative density as their phase compositions did not differ significantly.

**Figure 10:**
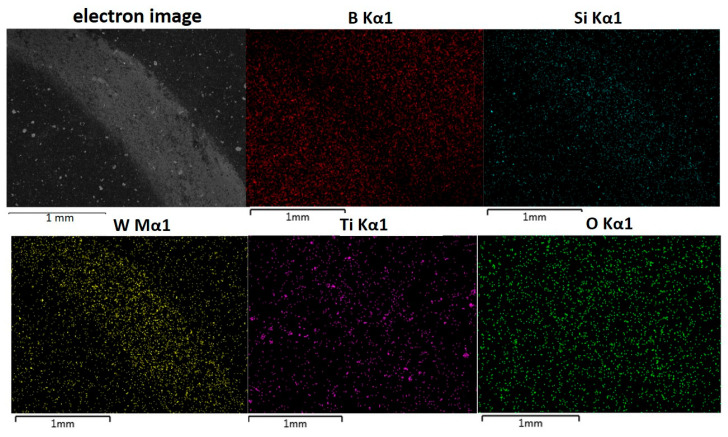
SEM image of the surface of B_4_C composite sintered at 1900 °C after friction-wear test (WC as counterpart material), with EDS maps showing distribution of B, Ti, Si, W, and O.%.

Microscopic observations of the friction paths of all composite samples indicate that the main mechanism of wear an abrasive type. Figure 10 shows the microstructure and EDS maps registered from the surface of composite sintered at 1900 °C, with a part of the track area worn in the friction-wear test. The EDS map for W indicates the intrusion of this element from the cooperating WC ball. The distribution of W, Ti, and Si related to the EDS map for O suggests the presence of their oxides. Tungsten oxides present in the worn area could have been formed during the test as products of tribochemical reactions. However, oxides in other areas were probably formed during sample storage.

## 4. Conclusions

Spark plasma sintering is a quick and effective method to densify boron carbide powders of stoichiometry B_13_C_2_ with the addition of 5% vol. Ti_5_Si_3_ to a relative density up to 85.2% of the theoretical one.

SPS sintering of the initial boron carbide powders with the addition of 5% vol. Ti_5_Si_3_ in temperatures 1700 °C–1900 °C results in the partial consumption of B_13_C_2_ and a complete disappearance of Ti_5_Si_3_ to form TiB_2_, SiC phases, and traces of carbon. The trace amounts of C most likely come from graphite elements and stamps used in the spark plasma sintering unit.

The Vickers hardness of the produced composites was in the range of 4–12 GPa and decreased with the increase in TiB_2_ content.

Resistance to wear of the produced boron carbide composites tested in the dry friction contact with WC as a counterpart material was better than that referred for B_4_C sinters. The main mechanism of wear was abrasive.

The average COF values of B_13_C_2_-TiB_2_-SiC composites sintered at 1800 °C and 1900 °C and tested in friction contact with WC were 0.54 and 0.58, respectively, i.e., slightly lower than the value reported for B_4_C sinters (0.6).

## Figures and Tables

**Table 1:** Particle size distribution in powder mixture after homogenization.

	μm		
	25%D	50%D	75%D
Mean Value	4.923	6.464	8.342
Std Dev.	0.042	0.059	0.104

**Table 2:** Phase composition and relative density of composites sintered by SPS from initial powder mixture of 95% B_4_C 5% vol. Ti_5_Si_3_.

SPS Temp/Time	ρ Theor. [%]	Phase Composition of Composite [wt.%]			
		B_13_C_2_	TiB_2_	SiC	C
1700 °C/10 min	76.5 ± 9.1	98	1	0.6	trace
1800 °C/10 min	81.5 ± 7.2	96	2.7	0.63	trace
1900 °C/10 min	85.2 ± 8.7	96	2.8	0.6	trace
ICCD (card code)		01-071-0108	04-010-8469	73-1665	04-015-2432

## Data Availability

The raw data supporting the conclusions of this article will be made available by the authors on request.
